# EXPLORING CIND STABILITY ACROSS TIME: INSIGHTS FOR IMPROVING COGNITIVE IMPAIRMENT CLASSIFICATIONS

**DOI:** 10.1093/geroni/igae098.3190

**Published:** 2024-12-31

**Authors:** Cynthia McDowell, Nicholas Tamburri, Jodie Gawryluk, Stuart MacDonald

**Affiliations:** University of Victoria, Victoria, British Columbia, Canada; University of Victoria, Victoria, British Columbia, Canada; University of Victoria, Victoria, British Columbia, Canada; University of Victoria, Victoria, British Columbia, Canada

## Abstract

**Objective:**

Cognitive impairment no dementia (CIND) classifications are susceptible to false positives and may not be indicative of progressive cognitive decline or dementia risk. The current project sought to investigate the dynamic nature of CIND across six years and to elucidate whether baseline impairment severity (single vs. multidomain impairment) predicts CIND stability patterns such as reversion and fluctuation between cognitive statuses. Method: Utilizing data from Project MIND, participants (N=259; 65-90 years) were classified as either Normal Cognition (NC) or CIND across five annual assessments using a battery of five cognitive tasks. Participants were classified as either Stable NC, Stable CIND, Progressors (NC—CIND), Reverters (CIND—NC) or Fluctuaters (CIND—NC—CIND) according to their across-time stability patterns. Participants initially classified as CIND were further subdivided based on impairment severity: single-task (CIND-S) or multiple-task (CIND-M) impairment. Multinomial logistic regression was utilized to explore whether baseline CIND severity differentiated Reverters and Fluctuaters from those who remained Stable CIND.

**Results:**

Most individuals were unstable in their CIND status for several years following baseline assessment. Compared to those classified as Stable CIND, Fluctuaters (-1.52, p=.01) and Reverters (-1.68, p=.01) were more likely to be classified as CIND-S (72% and 74%, respectively). Conclusions: Reverting back to NC or fluctuating between cognitive statuses is partly a function of instability in CIND-S classifications. Moving forward, CIND classifications could be improved through careful consideration of CIND severity at initial assessment. In particular, those classified as CIND-M may represent an at-risk group most likely to exhibit progressive cognitive impairment.

